# Peptidhormonanaloga basierte medikamentöse Adipositastherapie ist effektiv: Was ist die Evidenz zu harten Endpunkten und zum Langzeitverlauf?

**DOI:** 10.1007/s00104-025-02402-z

**Published:** 2025-11-05

**Authors:** Muzamil Hussain, Alexander Dimitry Miras

**Affiliations:** School of Medicine, Ulster University, Northland Rd, BT48 7JL Londonderry, Großbritannien

**Keywords:** Liraglutid, Semaglutid, Tirzepatid, Fettleibigkeit, Gewichtsabnahme, Liraglutide, Semaglutide, Tirzepatide, Obesity, Weight reduction

## Abstract

Seit nunmehr 10 Jahren stehen Medikamente auf der Basis anorektischer Peptidhormone zur Behandlung der Adipositas (OMM) zur Verfügung. In dieser Übersichtsarbeit wird die Evidenz zu den 3 behördlich zugelassenen und am häufigsten verwendeten Medikamenten (Liraglutid, Semaglutid und Tirzepatid) dargestellt. In diesem Kontext wurde innerhalb des SCALE-Programms (Liraglutid), des STEP-Programm (Semaglutid) und des SURMOUNT-Programms (Tirzepatid) eine Vielzahl von hochwertigen placebokontrollierten prospektiv randomisierten Studien durchgeführt. Demnach liegt der Wirkungsgrad auf die mittlere Gewichtsabnahme für Liraglutid bei 6–8 %, für Semaglutid bei 9,6–15 % und für Tirzepatid bei 14,7–22,1 %. Ebenso zeigen die Medikamente einen hohen antidiabetischen Wirkungsgrad, wobei die erreichte Gewichtsabnahme bei Diabetikern meist geringer ist. Insgesamt sind die Sicherheit und Verträglichkeit der 3 Medikamente hoch. Die häufigsten unerwünschten Ereignisse treten meist früh während der Behandlung auf und klingen mit der Zeit ab. Die positiven Effekte werden durch ein Absetzen der Therapie meist nivelliert. Bezüglich harter Endpunkte konnte die SELECT-Studie für schwere unerwünschte kardiovaskuläre Ereignisse (MACE) eine Risikoreduktion von 20 % unter Semaglutid zeigen. Daten zu weiteren harten Endpunkten und zum Langzeitverlauf existieren bisher nicht.

**Zusatzmaterial online:**

In der Online-Version dieses Artikels (10.1007/s00104-025-02402-z) sind 3 weitere Tabellen enthalten.

## Abstract (en)

The anorectic peptide-based obesity management medications (OMM) have dominated the treatment of obesity over the last decade. This review analyses the evidence that has laid the ground for the three most frequently used drugs (liraglutide, semaglutide and tirzepatide) with regulatory approval. In this context, the SCALE program investigated liraglutide for weight loss and found its effectiveness to be roughly 6–8%. Similarly, the STEP program extensively researched semaglutide as a weight loss intervention and found it to be effective in reducing body weight by 9.6% in people with type 2 diabetes and by 15% in those without. Likewise, for tirzepatide this evaluation was done under the SURMOUNT program, which reported an efficacy of 14.7% in body weight reduction for type 2 diabetes patients as opposed to 22.1% in people without diabetes. The positive effects usually diminish when treatment is discontinued. The SELECT trial provided the first concrete evidence of risk reduction of major adverse cardiovascular events (MACE) by 20% with the use of semaglutide. Overall, safety and tolerability of these 3 drugs is generally good across all studies. Additionally, the most common adverse events reported are gastrointestinal, which occur early during treatment and subside over time. Data on other hard end points and long-term outcomes do not yet exist.

Die konservative Behandlung der Adipositas wird derzeit von Substanzklassen, die auf anorektischen Peptidenterohormonen basieren, dominiert. Die bisherigen Medikamente enthalten Glukagon-ähnliche Rezeptor-1-Agonisten (GLP1RA) und das gastrische inhibitorische Polypeptid (GIP). Diese Substanzen werden bisher als Monotherapie wie bei Liraglutid (GLP1RA) und Semaglutid (GLP1RA) oder kombiniert wie bei Tirzepatid (GLP1RA + GIP) eingesetzt. Weitere Substanzklassen und Kombinationspräparate sind in klinischer Erprobung.

In den Vereinigten Staaten wurden allein im Februar 2024 1,5 Mio. Rezepte zur medikamentösen Adipositastherapie ausgestellt, wobei Semaglutid, Liraglutid und Tirzepatid die am häufigsten verschriebenen Medikamente waren [1]. Dabei wird nur ein Bruchteil der Patienten mit Adipositas wirksam behandelt. So sind weltweit ca. 880 Mio. Erwachsene adipös, wobei die Prävalenz weiter steigt [2]. Damit geht eine steigende Zahl von Adipositas-assoziierten Herz-Kreislauf-Erkrankungen einher, die weltweit bei ca. 612 Mio. Fällen liegt, diese machen den Hauptanteil (26,8 %) der weltweiten vorzeitigen Todesfälle aus [3].

Gemäß der Lancet-Kommission [4] und der EASO(European Association for the Study of Obesity)-Leitlinien [5] sollen daher die Adipositas und die damit einhergehenden Herz-Kreislauf-Erkrankungen frühzeitiger und aggressiver behandelt werden.

In dieser Übersicht wird die bisherige Evidenz zur Pharmakotherapie der Adipositas dargestellt. Dabei liegt der Schwerpunkt auf hochwertigen Studien, die die Grundlage für die aktuellen klinischen Leitlinien und behördlichen Zulassungen bilden. Die in dieser Übersicht behandelten Forschungsprogramme und Studien zu Liraglutid, Semaglutid und Tirzepatid umfassen die sog. SCALE-, STEP-, SELECT- und SURMOUNT-Programme.

## Liraglutid

Um die Wirksamkeit und Sicherheit von Liraglutid zur Behandlung der Adipositas zu evaluieren, wurde das SCALE-Programm initiiert. Die Ergebnisse dieses Programms führten zur behördlichen Zulassung von Liraglutid und bildeten die Grundlage für evidenzbasierte Therapieempfehlungen.

### Wirksamkeit bei Adipositas und Prädiabetes

In der ersten placebokontrollierten randomisierten Studie (SCALE 1) wurde untersucht, ob Liraglutid (3 mg 1‑mal täglich subkutan) zusammen mit regelmäßigen Ernährungsberatungen zu einer relevanten Gewichtsabnahme führt [6].

In dieser placebokontrollierten prospektiv randomisierten Studie lag der Frauenanteil bei 78 %, das Durchschnittsalter bei 45,2 Jahren, der Durchschnitts-BMI (Body Mass Index) bei 38,3 kg/m^2^ und der mittlere HbA_1c_-Wert bei 5,6 % [6]. Nach 56 Wochen verlor die Liraglutid-Gruppe im Mittel 8,0 % ihres Körpergewichts (vs. 2,6 % für Placebo) [6]. Die Liraglutid-Gruppe wies gegenüber der Placebokohorte auch einen höheren Prozentsatz an Teilnehmern auf, die ≥ 5 % ihres Körpergewichts reduzieren konnten (63,2 vs. 27,1 %) (Tabelle e1 im Online-Supplement) [6]. Interessanterweise war das Ansprechen auf die Behandlung bei allen Teilnehmern unabhängig eines Prädiabetes und über alle BMI-Kategorien hinweg ähnlich. Das Auftreten eines Typ-2-Diabetes war in der Liraglutid- im Vergleich zur Placebogruppe deutlich geringer [6]. Insgesamt erwies sich Liraglutid gegenüber Placebo hinsichtlich der Gewichtsabnahme, der Blutzuckerkontrolle, der kardiometabolischen Risikofaktoren und der gesundheitsbezogenen Lebensqualität überlegen.

### Adipositas und Typ-2-Diabetes mellitus

Um die Wirksamkeit von Liraglutid bei Typ-2-Diabetikern zu untersuchen, wurden die Studien SCALE 2 und SCALE 3 durchgeführt. In der SCALE 2-Studie wurden zunächst nicht Insulin-pflichtige Diabetiker eingeschlossen [7]. Diese doppelblinde Studie umfasste 3 Gruppen (Liraglutid 3,0 mg vs. Liraglutid 1,8 mg vs. Placebo). Zusätzlich erhielten alle Patienten wie in SCALE 1 eine Lebensstilintervention. Nach 56 Wochen betrug der durchschnittliche Gewichtsverlust 6,0 % für Liraglutid (3,0 mg), 4,7 % für Liraglutid (1,8 mg) und 2,0 % für die Placebogruppe (Tabelle e1 im Online-Supplement) [7]. Ein Gewichtsverlust von ≥ 5 % wurde von 54,3 % der Teilnehmer in der Liraglutid‑3,0‑mg-Gruppe, 40,4 % in der Liraglutid‑1,8‑mg-Gruppe und lediglich 21,4 % in der Placebogruppe erreicht (Tabelle e1 im Online-Supplement) [7].

Die anschließend durchgeführte SCALE 3-Studie schloss insulinpflichtige Diabetiker ein [8]. Die Gruppen erhielten entweder Liraglutid (3,0 mg) oder Placebo sowie eine additive intensive Verhaltensintervention. Nach 56 Wochen betrug der durchschnittliche Gewichtsverlust 5,8 % in der Liraglutid-Gruppe gegenüber 1,5 % in der Placebogruppe [8]; 51,8 % der Liraglutid-Kohorte erreichten einen Gewichtsverlust von ≥ 5 %, verglichen mit 24,0 % in der Placebogruppe [8]. Darüber hinaus war Liraglutid bei der Verbesserung des HbA_1c_ überlegen (Tabelle e1 im Online-Supplement) [8].

### Aufrechterhaltung der Gewichtsabnahme

In der SCALE Maintenance-Studie wurde untersucht, ob eine rein diätetisch erzielte Gewichtsabnahme durch Liraglutid (3,0 mg) aufrechterhalten werden kann [10]. Nach einer 12-wöchigen Diät wurden diejenigen Patienten, die ≥ 5 % ihres Gewichts verloren hatten, entweder einer Liraglutid‑3,0‑mg- oder Placebogruppe zugeteilt. Während der folgenden 56-wöchigen Behandlungsphase verlor die Liraglutid-Gruppe zusätzlich 6,2 % ihres Körpergewichts, während die Placebogruppe ein Defizit von 0,2 % erreichte [10] (Tabelle e1 im Online-Supplement); 81,4 % der Liraglutid-Gruppe vs. 48,9 % der Placebogruppe behielten den durch die initiale Diät erzielten Gewichtsverlust von ≥ 5 % [10]. Beide Gruppen zeigten jedoch während der 12-wöchigen Nachbeobachtungszeit ohne eine weitere Behandlung einen Wiederanstieg des Körpergewichts [10].

Eine moderate Wirksamkeit von Liraglutid auf die Gewichtsreduktion konnte gezeigt werden

Zusammenfassend konnte in hochrangigen Studien eine moderate Wirksamkeit von Liraglutid auf die Gewichtsreduktion gezeigt werden. Ebenfalls konnten präventive Effekte bei einem Prädiabetes (SCALE 1) und eine gute Wirksamkeit auf einen bestehenden Typ-2-Diabetes (SCALE 2 und SCALE 3) nachgewiesen werden, ebenso die Effektivität auf eine Gewichtsstabilisierung nach vorangegangener Diät (SCALE 5). Die therapeutische Wirkung bestand allerdings nur, solange das Medikament appliziert wurde. Daten zu harten Endpunkten liegen hingegen nicht vor.

## Semaglutid

Um die Wirksamkeit und Sicherheit von Semaglutid zur Adipositasbehandlung zu validieren, wurde das STEP-Programm initiiert.

### Wirksamkeit bei Adipositas und Prädiabetes

Zuerst wurde in der placebokontrollierten prospektiv randomisierten Studie STEP 1 [11] die Wirksamkeit von Semaglutid (2,4 mg wöchentlich) auf die Gewichtsreduktion an nichtdiabetischen übergewichtigen oder adipösen Erwachsenen untersucht. Beiden Gruppen wurde zusätzlich eine Lebensstilintervention angeboten. Nach 68 Wochen betrug der durchschnittliche Gewichtsverlust mit Semaglutid 14,9 % gegenüber 2,4 % bei Placebo (Tabelle e2 im Online-Supplement) [11]. Einen Gewichtsverlust von ≥ 10 % erreichten 69,1 % vs. 12,0 %, einen von ≥ 15 % 50,5 % vs. 4,9 %, und einen von ≥ 20 % erreichten 32,0 % vs. 1,7 % [11]. Eine Blutzuckernormalisierung wurde in 84,1 % unter Semaglutid erreicht [11]. Nach Abschluss der 68-wöchigen Interventionen wurde in der STEP 1-Verlängerungsstudie für insgesamt 120 Wochen ohne fortgesetzte Therapie weiter nachbeobachtet [12]. Am Ende des Studienzeitraums waren sämtliche therapeutische Effekte nivelliert (Tabelle e2 im Online-Supplement).

Eine weitere Studie, die die Auswirkungen des Absetzens von Semaglutid untersuchte, war STEP 4 [13]. Hierin wurden die Patienten 20 Wochen mit Semaglutid (2,4 mg wöchentlich) behandelt und anschließend für 48 Wochen weiterbehandelt oder auf Placebo gesetzt. Unter Placebo waren nach 68 Wochen die initial positiven Auswirkungen von Semaglutid auf gewichtsbezogene Gesundheitsparameter nivelliert, während unter fortgesetzter Therapie mit Semaglutid weitere Verbesserungen evident waren (s. Tabelle e2 im Online-Supplement).

### Adipositas und Typ-2-Diabetes mellitus

In der Studie STEP 2 wurden nun nicht Insulin-pflichtige Diabetiker eingeschlossen [14]. Hier wurde auch die Dosis-Wirkungs-Beziehung von Semaglutid (2,4 mg vs. 1,0 mg vs. Placebo) auf die Gewichtsreduktion über einen Zeitraum von 68 Wochen untersucht. Am Ende der Studie war Semaglutid (2,4 mg vs. 1,0 mg) überlegen [14]. Darüber hinaus erreichten mehr Personen mit Semaglutid 2,4 mg (68,8 %) einen Gewichtsverlust von ≥ 5 % als mit Semaglutid 1,0 mg (57,1 %) oder Placebo (28,5 %). Ebenso erzielte die höhere Dosis auf den Typ-2-Diabetes einen höheren Wirkungsgrad (Tabelle e2 im Online-Supplement). Diese Ergebnisse konnten in STEP 6 in einer asiatisch-pazifischen Bevölkerung und in STEP 7 bestätigt werden [15].

### Ergänzung zu intensiven Lebensstilinterventionen

In der STEP 3-Studie [17] wurde die Wirksamkeit von Semaglutid während einer intensiven Lebensstilintervention überprüft. Die eingeschlossenen nichtdiabetischen Personen erhielten entweder Semaglutid (2,4 mg wöchentlich) oder Placebo. Nach 68 Wochen konnte Semaglutid im Vergleich zum Placebo eine zusätzliche Gewichtsabnahme von 10,3 % erzielen [17]. Ebenso war die Wahrscheinlichkeit, die Gewichtsabnahmeziele von ≥ 5 %, ≥ 10 % und ≥ 15 % des Ausgangsgewichts zu erreichen, mit Semaglutid weitaus größer als mit Placebo (Tabelle e2 im Online-Supplement).

### Vergleich zwischen Semaglutid und Liraglutid

In STEP 8 wurde die Effektivität von Semaglutid (2,4 mg wöchentlich) und Liraglutid (3,0 mg täglich) über 68 Wochen direkt verglichen [19]. Die Mehrheit der Teilnehmenden waren Frauen (78,4 %), das mittlere Alter lag bei 49 Jahren und der BMI bei 37,5 kg/m^2^, wobei 36,1 % einen Prädiabetes hatten. Unter Semaglutid wurde ein um 9,4 % höherer Körpergewichtsverlust erreicht (Tabelle e2 im Online-Supplement) [19].

### Adipositas und Prädiabetes

In STEP 10 wurde die Effektivität von Semaglutid zur Behandlung eines Prädiabetes bei übergewichtigen oder adipösen Patienten untersucht [21]. Am Ende der 52-wöchigen Behandlungsdauer zeigte Semaglutid eine überlegene Reduktion des Körpergewichts und einen höheren Anteil an Teilnehmern, die eine Normoglykämie erreichten (81,0 % vs. 14 %) (Tabelle e2 im Online-Supplement).

Semaglutid hat einen hohen Wirkungsgrad auf die Gewichtsreduktion bei adipösen Patienten

Zusammenfassend konnte das STEP-Programm einen hohen Wirkungsgrad von Semaglutid auf die Gewichtsreduktion (10 bis 17 %) bei adipösen Patienten ohne (STEP 1) und mit Diabetes (STEP 2, STEP 6 und STEP 7) zeigen. Dabei war Semaglutid Liraglutid überlegen (STEP 8). Der Gewichtverlust wurde im Wesentlichen während des ersten Jahres erreicht und stabilisierte sich hiernach unter fortdauernder Gabe (STEP 1 und STEP 5), ein Absetzten nivellierte die erreichten Effekte. Subjektive und funktionelle Verbesserungen bei Kniegelenkarthrose konnten ebenfalls gezeigt werden (STEP 9).

## Tirzepatid

Um die Wirksamkeit und Sicherheit von Tirzepatid zur Adipositasbehandlung zu untersuchen, wurde das SURMOUNT-Programm durchgeführt.

### Wirkungsgrad bei Adipositas und Prädiabetes

Als Meilensteinstudie dieses Programms wird SURMOUNT 1 angesehen [22]. Diese Studie war zweiphasig konzipiert. Patienten ohne einen Prädiabetes wurden für 72 Wochen beobachtet, während Patienten mit Prädiabetes in einer 2‑jährigen Verlängerungsphase weiter beobachtet wurden. Die Patienten wurden in 4 Gruppen (5 mg vs. 10 mg vs. 15 mg Tirzepatid vs. Placebo) eingeteilt. Nach 72 Wochen zeigten alle Tirzepatid-Dosierungen einen signifikant höheren Gewichtsverlust im Vergleich zu Placebo, wobei der therapeutische Unterschied gegenüber Placebo dosisabhängig war (Tab. 1; [22]). Während der 2‑jährigen Verlängerungsphase (insgesamt 176 Wochen) wurden die mittelfristige Gewichtsabnahme und die Prävalenz eines Typ-2-Diabetes bei einer prädiabetischen Population untersucht. Nach 176 Wochen lag die Gewichtsabnahme gegenüber Placebo weiterhin ähnlich (für 5 mg bei 11,1 %; für 10 mg bei 17,5 % und für 15 mg bei 18,4 % (Tab. 1; [23])). Ein Typ-2-Diabetes trat bei 1,3 % in der Tirzepatid-Gruppe (alle Dosierungen) und bei 13,3 % in der Placebogruppe auf (Tab. 1; [23]). Ebenso erreichten mehr Probanden in den Tirzepatid-Gruppen eine Normoglykämie, wobei das Erreichen der Normoglykämie mit dem Ausmaß der Gewichtsabnahme korrelierte [23].

**Tab. 1:** SURMOUNT-Programm (Studien und Ergebnisse für Tirzepatid)

*Studie*	*Ergebnisse nach 72 Wochen*	*Tirzepatid (5* *mg), n* *=* *630*	*Tirzepatid (10* *mg), n* *=* *636*	*Tirzepatid (15* *mg) n* *=* *630*	*,Placebo, n* *=* *643*
		*Mittelwerte und (95* *% CI)*			
*SURMOUNT 1 Prädiabetes [*22*]*	Gewichtsverlust in %	−15,0 (−15,9 bis −14,2)	−19,5 (−20,4 bis −18,5)	−20,9 (−21,8 bis −19,9)	−3,1 (−4,3 bis −1,9)
	Unterschied zum Placebo in % (95 % CI)	−11,9 (−13,4 bis −10,4)	−16,4 (−17,9 bis −14,8)	−17,8 (−19,3 bis −16,3)	–
	Gewichtsverlust von ≥10 % in %	85,1 (81,6 bis 88,6)	88,9 (85,9 bis 91,9)	90,9 (88,0 bis 93,8)	34,5 (29,8 bis 39,2)
*Studie*	*Ergebnisse nach 176 Wochen*	*Tirzepatid (5* *mg), n* *=* *247*	*Tirzepatid (10* *mg), n* *=* *262*	*Tirzepatid (15* *mg), n* *=* *253*	*Placebo, n* *=* *270*
*SURMOUNT 1, Verlängerung (3 Jahre) [*23*]*	Gewichtsverlust in % (95 % CI)	−12,3 (−14,5 bis −10,2)	−18,7 (−24,1 bis −13,4)	−19,7 (−22,1 bis –17,3)	−1,3 (−4,0 bis –1,5)
	Unterschied zum Placebo in % (95 % CI)	−11,1 (−14,4 bis −7,8)	−17,5 (−23,6 bis −11,3)	−18,4 (−22,2 bis −14,7)	–
	Neue Fälle mit Typ-2-Diabetes (%)	Alle Tirzepatid-Gruppen			36 (13,3)
		10 (1,3)			
	HR, für alle Tirzepatid-Gruppen vs. Placebo (95 % CI)	Alle Tirzepatid-Gruppen			–
		0,07 (0,0 bis 0,1)			
*Studie*	*Ergebnisse nach 72 Wochen*	*Tirzepatid (10* *mg), n* *=* *312*		*Tirzepatid (15* *mg), n* *=* *311*	*Placebo, n* *=* *315*
*SURMOUNT 2 [*25*]*	Gewichtsverlust in % (SD)	−12,8 (0,6)		−14,7 (0,5)	−3,2 (0,5)
	ETD zu Placebo (95% CI), *p*-Wert	−9,6 (−11,1 bis −8,1), *p* < 0,0001		−11,6 (−13,0 bis −10,1), *p* < 0,0001	–
	Gewichtsverlust von ≥5 % in %	79 %		83 %	32 %
	Odds Ratio gegenüber Placebo (95% CI), *p*-Wert	8,3 (5,6 bis 12,3), *p* < 0,0001		10,5 (6,8 bis 161), *p* < 0,0001	–
*Studie*	*Ergebnisse*		*Tirzepatid (10* *mg oder 15* *mg)*		*Placebo*
*SURMOUNT 3 Erhalt der Gewichtsabnahme [*26*]*	*12-Wochen Diät*		*n* *=* *287*		*n* *=* *292*
	Ausgangsgewicht in kg		110,1 (23,9)		108,9 (22,2)
	Veränderung durch Diät in kg oder %		−7,6 (2,9) kg		−7,6 (2,8) kg
			−6,9 (1,9) %		−7,0 (2,0) %
	*Ergebnisse nach 72 Wochen Tirzepatid vs. Placebo*		*n* *=* *287*		*n* *=* *292*
	Körpergewicht nach Diät (kg) vor Pharmakointervention		102,5 (22,1)		101,3 (20,7)
	Gewichtsveränderung in % (SE)		−18,4 (0,7)		2,5 (1,0)
	ETD zum Placebo (95% CI), *p*-Wert		−20,8 (−23,2 bis −18,5), *p* < 0,001		–
	Gewichtsverlust von ≥5 % in %		87,5 (2,2)		16,5 (3,0)
	Odds Ratio gegenüber Placebo (95% CI), *p*-Wert		34,6 (19,2 bis 62,6), *p* < 0,001		–
*Studie*	*Ergebnisse nach 88 Wochen (95* *% CI)*		*Tirzepatid (10* *mg oder 15* *mg), n* *=* *335*		*Placebo, n* *=* *335*
*SURMOUNT 4 Medikation zur Stabilisierung nach Diät [*27*]*	Ausgangsgewicht in kg		107,3 (22,3)		
	Körpergewicht nach 36 Wochen		84,6 (19,8)		85,8 (22,3)
	Veränderung bis Woche 36 in %		−5,5 (−6,8 bis −4,2)		14,0 (12,8 bis 15,2)
	Veränderung bis Woche 36 in kg (95 % CI), *p*-Wert		−19,4 (−21,2 bis −17,7), *p* < 0,001		–
	Gewichtsveränderungen von Woche 36 zu Woche 88 in kg		−4,7 (−5,7 bis −3,6), *p* < 0,001		11,1 (10,1 bis 12,2)
	Unterschied (95 % CI), *p*-Wert		−15,8 (−17,3 bis −14,3), *p* < 0,001		–
	Anteil derer, die von Woche 36 bis Woche 88 ≥ 80 % des initial erreichten Gewichtsverlustes hielten (%)		300 (89,5)		55 (16,6)
	Unterschied (95 % CI), *p*-Wert		44,0 (24,9 bis 77,5), *p* < 0,001		–
*Studie*	*Ergebnisse nach 72 Wochen*		*Tirzepatid (10* *mg oder 15* *mg), n* *=* *374*		*Semaglutid (1,7* *mg oder 2,4* *mg), n* *=* *376*
*SURMOUNT 5 Vergleich von Semaglutid mit Tirzepatid [*28*]*	Ausgangsgewicht in kg (SD)		112,7 ± 24,8		113,4 ± 26,3
	Gewichtsverlust in % (95 % CI)		−20,2 (−21,4 bis −19,1)		−13,7 (−14,9 bis −12,6)
	Behandlungsunterschied (95% CI)		−6,5 (−8,1 bis −4,9)		
	Gewichtsverlust in kg (95% CI)		−22,8 (−24,1 bis −21,5)		−15,0 (−16,3 bis −13,7)
	Behandlungsunterschied (95% CI)		−7,9 (−9,7 bis −6,0)		
	Gewichtsverlust von ≥10 % (%)		304 (81,6)		227 (60,5)
	Relatives Risiko (95% CI)		1,3 (1,2 bis 1,5)		
	Gewichtsverlust von ≥15 % (%)		241 (64,6)		151 (40,1)
	Relatives Risiko (95% CI)		1,6 (1,4 bis 1,9)		
	Gewichtsverlust von ≥20 % (%)		181 (48,4)		103 (27,3)
	Relatives Risiko (95% CI)		1,8 (1,5 bis 2,2)		

*CI* Konfidenzintervall, *SE* Standard Error of the Mean -> Standardfehler des Mittelwerts

### Wirkungsgrad bei Adipositas und Typ-2-Diabetes mellitus

Nachfolgend wurde in der SURPASS-2-Studie der Wirkungsgrad von Tirzepatid bei nicht Insulin-pflichtigen Typ-2-Diabetikern untersucht [25]. Die Teilnehmer erhielten neben einer Lebensstilintervention Tirzepatid 10 mg vs. Tirzepatid 15 mg vs. Placebo. Am Ende der 72 Wochen betrug die durchschnittliche Gewichtsveränderung gegenüber dem Ausgangswert 12,8 % für 10 mg, 14,7 % für 15 mg gegenüber 3,2 % für Placebo (Tab. 1; [25]).

### Gewichtserhaltung nach intensiver Lebensstilintervention

Analog der SCALE Maintenance-Studie wurde die Effektivität von Tirzepatid auf die Aufrechterhaltung einer rein diätetisch erzielten Gewichtsabnahme in SURMOUNT 3 untersucht [26]. Nach einer 12-wöchigen Diät wurden diejenigen, die ≥ 5 % ihres Gewichts reduziert hatten, einer Tirzepatid oder Placebogruppe zugeteilt. Die initiale 12-wöchige Lebensstilintervention führte zu einem Gewichtsverlust von 6,9 % des mittleren Ausgangsgewichts ([26]; Tab. 1). Nach 72 Wochen betrug der Gewichtsverslust zum Zeitpunkt der Randomisierung weitere 18,4 % für Tirzepatid gegenüber einer Zunahme von 2,5 % für Placebo (Tab. 1; [26]).

### Langfristige Gewichtskontrolle

In SURMOUNT 4 wurde untersucht, ob eine fortdauernde Gabe von Tirzepatid die initial erzielte Gewichtsabnahme aufrechterhalten kann [27]. In dieser Studie wurden die Teilnehmer zunächst 36 Wochen lang mit Tirzepatid behandelt, dann in eine Tirzepatid- oder Placebogruppe randomisiert. Nach 88 Wochen betrug der durchschnittliche Gewichtsverlust in der Tirzepatid-Gruppe 5,5 % gegenüber einer Gewichtszunahme von 14,0 % bei Placebo (Tab. 1; [27]). Darüber hinaus gelang es 89,5 % der Tirzepatid- gegenüber 16,6 % der Placebogruppe, mindestens 80 % des in den ersten 36 Wochen verlorenen Gewichts zu halten (Tab. 1; [27]).

### Vergleich von Tirzepatid mit Semaglutid

In SURMOUNT 5 erfolgte dann der direkte Vergleich der Effektgröße von Tirzepatid und Semaglutid [28]. In dieser Studie wurden Patienten mit Übergewicht oder Adipositas ohne Diabetes entweder in die Tirzepatid-Gruppe oder in die Semaglutid-Gruppe randomisiert. Nach 72 Wochen betrug die Gewichtsreduktion in der Tirzepatid-Gruppe 20,2 % gegenüber 13,7 % in der Semaglutid-Gruppe, wobei der therapeutische Unterschied mit 6,5 Prozentpunkten signifikant war (Tab. 1; [28]).

Das SURMOUNT-Programm konnte den sehr hohen Wirkungsgrad von Tirzepatid nachweisen

Zusammenfassend konnte das SURMOUNT-Programm den sehr hohen Wirkungsgrad (Gewichtsverlust von 14,7–22,1 %) von Tirzepatid nachweisen. Dabei wurde gezielt die Wirksamkeit bei Personen ohne (SURMOUNT 1) und mit Typ-2-Diabetes (SURMOUNT 2) untersucht. Gleichwohl wurden die Prävention eines Typ-2-Diabetes mellitus (T2DM) und die Aufrechterhaltung einer Gewichtsreduktion über einen Zeitraum von 3 Jahren (SURMOUNT 1, 2‑jährige Verlängerung), die Gewichtserhaltung nach intensiver Lebensstilintervention (SURMOUNT 3) und nach initialer Behandlung mit Tirzepatid selbst (SURMOUNT 4) evaluiert. Schließlich konnte die Überlegenheit gegenüber Semaglutid (SURMOUNT 5) nachgewiesen werden.

#### Kardiovaskuläre Ereignisstudien (CVOT)

Die randomisierte doppelblinde placebokontrollierte SELECT-Studie konnte für die Therapie mit Semaglutid eine Reduktion von schwerwiegenden kardiovaskulären Ereignissen (MACE) zeigen [29]. Als Teilnehmer wurden Patienten mit einer nachgewiesenen kardiovaskulären Erkrankung ohne Diabetes und mit einem BMI von ≥ 27,0 kg/m^2^ aufgenommen. Die Mehrzahl der Teilnehmer war männlich (72,3 %), das Durchschnittsalter lag bei 61,6 Jahren, der durchschnittliche BMI bei 33,3 kg/m^2^, der mittlere HbA_1c_-Wert bei 5,8 ± 0,3 %, wobei 66,4 % im Prädiabetesbereich lagen. Die durchschnittliche Behandlungsdauer mit Semaglutid betrug etwa 33 Monate. In der Semaglutid-Gruppe traten bei 6,5 % vs. 8,0 % (Placebo) der Teilnehmer primäre kardiovaskuläre Ereignisse (Tod durch kardiovaskuläre Ursachen, nichttödlicher Myokardinfarkt oder nichttödlicher Schlaganfall) auf [29]. Somit senkte Semaglutid das Risiko für kardiovaskuläre Ereignisse in diesem Zeitraum signifikant um 20 % (Tab. 2).

**Tab. 2:** Studienergebnisse zur Risikoreduktion kardiovaskulärer Ereignisse durch Semaglutid-Behandlung

Studie	Ergebnisse	Semaglutid 2,4 mg, *n* = 8803	Placebo, *n* = 8801	Hazard Ratio (95 % CI)	*p*-Wert
		N (%)	N (%)		
*SELECT Semaglutide* [29]	Primärer kardiovaskulärer Endpunkt (Tod durch kardiovaskuläre Erkrankung, nichttödlicher Herzinfarkt oder Schlaganfall)	569 (6,5)	701 (8,0)	0,80 (0,72–0,90)	< 0,001
	Tod durch kardiovaskuläre Erkrankung	223 (2,5)	262 (3,0)	0,85 (0,71–1,01)	0,07
	Herzinsuffizienz	300 (3,4)	361 (4,1)	0,82 (0,71–0,96)	N/A
	Tod (gesamt)	375 (4,3)	458 (5,2)	0,81 (0,71–0,93)	N/A
	Erweiterter kardiovaskulärer Endpunkt (primärer Endpunkt plus koronare Revaskularisierung/Krankenhausaufenthalt wegen instabiler Angina)	873 (9,9)	1074 (12,2)	0,80 (0,73–0,87)	N/A
	Gemeinsamer kardiovaskulärer Endpunkt (Tod [gesamt] und nichttödlicher Herzinfarkt oder Schlaganfall)	710 (8,1)	877 (10,0)	0,80 (0,72–0,88)	N/A
	Nichttödlicher Herzinfarkt	234 (2,7)	322 (3,7)	0,72 (0,61–0,85)	N/A
	Nichttödlicher Schlaganfall	154 (1,7)	165 (1,9)	0,93 (0,74–1,15)	N/A
	Endpunkt Nephropathie (Tod durch renale Erkrankung, Dialyse oder Nierentransplantation), GFR niedriger 15/ml/min/1,73 m^2^, Reduktion der Ausgangs-GFR um > 50 %, persistierende Makroalbuminurie	155 (1,8)	198 (2,2)	0,78 (0,63–0,96)	N/A

*CI* Konfidenzintervall, *N/A* nicht angegeben, *GFR* glomeruläre Filtrationsrate

#### Sicherheit und Verträglichkeit der Pharmakotherapie

**Abb. 1:**
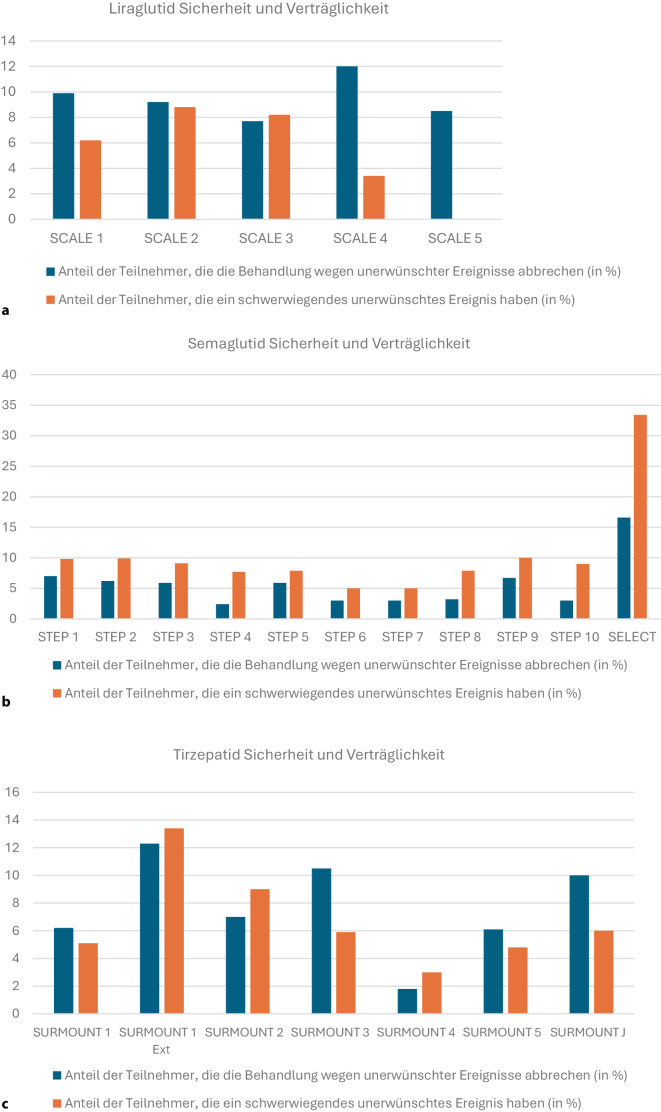
Sicherheit und Verträglichkeit. Liraglutid (**a**), Semaglutid (**b**) und Tirzepatid (**c**); „serious adverse event“ (SAE) gilt als Maß für die Sicherheit, der Behandlungsabbruch aufgrund von Nebenwirkungen als Maß für die Verträglichkeit

Im SCALE-Programm lag die Abbruchrate der Liraglutid-Behandlung aufgrund unerwünschter Ereignisse zwischen 7,7 und 12 %, die Inzidenz schwerwiegender unerwünschter Ereignisse zwischen 0 und 8,8 % (Abb. 1a; [6–10]). Insgesamt waren die meisten unerwünschten Ereignisse (UE) leicht oder mittelschwer. Die meist gastrointestinalen Symptome waren dosisabhängig und traten zumeist während der Dosistitration in den ersten Monaten der Behandlung auf (Tabelle e3 im Online-Supplement). Die aktuell diskutierten schweren unerwünschten Ereignisse wie Cholelithiasis, Cholezystitis, Pankreatitis, medulläres Schilddrüsenkarzinom und Suizidalität waren in allen SCALE-Studien sehr selten (Tabelle e3 im Online-Supplement).

Die Sicherheit und Verträglichkeit von Semaglutid (STEP-Programm) waren vergleichbar (Abb. 1b, Tabelle e3 im Online-Supplement) [11, 13–21]. Einige Studien berichteten über das Auftreten von Augenerkrankungen (diabetische Retinopathie, Netzhauterkrankungen). Die SELECT-Studie [29], die etwa 40 Monate dauerte, wies eine höhere Inzidenz von schwerwiegenden unerwünschten Ereignissen auf als das STEP-Programm (Abb. 1b).

Die anhand unerwünschter Ereignisse dokumentierten Behandlungsabbrüche für Tirzepatid lagen zwischen 1,8 und 12,3 %, die Sicherheit, gemessen am Auftreten schwerwiegender unerwünschter Ereignisse, lag zwischen 3 und 13,4 % (Abb. 1c; [22–28]). Es ist anzumerken, dass die SURMOUNT 1-Verlängerungsstudie eine höhere Häufigkeit sowohl von Behandlungsabbrüchen als auch von schwerwiegenden unerwünschten Ereignissen aufwies, da sie mit einer Dauer von 3 Jahren länger war als alle anderen Studien in diesem Programm.

## Diskussion und Einordnung

Hochrangige Studien konnten zeigen, dass nun wirksame und sichere Pharmakotherapien zur Behandlung der Adipositas zur Verfügung stehen. Alles deutet bisher darauf hin, dass die Medikamente dauerhaft eingenommen werden müssen, um die Therapiewirkung aufrechtzuerhalten. Bisher ist es nicht klar, ob und zu welchem Zeitpunkt die Medikamente abgesetzt werden können. In diesem Kontext müssen Daten zum Nebenwirkungsprofil und zur Häufigkeit bei langfristiger Einnahme weiter erhoben werden. Bisher existieren noch Evidenzlücken zum Wirkungsgrad bei extremer Adipositas, zur langfristigen Wirksamkeit und zu harten Endpunkten in Bezug auf viele Adipositas-assoziierte Erkrankungen sowie auf die Lebensqualität und das Gesamtüberleben. Aktuelle Daten deuten darauf hin, dass die metabolische bariatrische Chirurgie im direkten Vergleich zu GLP-1-Rezeptoragonisten nicht nur im Hinblick auf die erreichte Gewichtsabnahme, sondern auch im Hinblick auf mikro- und makrovaskuläre Komplikationen bisher überlegen ist [30, 31].

## Fazit für die Praxis

- Der mittlere Gewichtsverlust durch Liraglutid liegt bei 6–8 %, durch Semaglutid bei 9,6–15 % und durch Tirzepatid bei 14,7–22,1 %.
- Bei gleichzeitig hohem antidiabetischem Wirkungsgrad erreichen Diabetiker meist einen geringeren Gewichtsverlust.
- Das Absetzen der Medikamente führt kurzfristig zu einer Nivellierung der Effekte.
- Insgesamt sind die Medikamente gut verträglich bei hoher Sicherheit.
- Seltene nachgewiesene Nebenwirkungen sind eine Cholezystolithiasis, Cholezystitis und eine Pankreatitis. Eine suszipierte erhöhte Rate an diabetischer Retinopathie, psychiatrischen Erkrankungen und dem medullären Schilddrüsenkarzinom ist nicht ausreichend bewiesen.
- Die SELECT-Studie hat gezeigt, dass die langfristige Anwendung von Semaglutid das Risiko für Herz-Kreislauf-Erkrankungen um 20 % senken kann.
- Bisher gibt es keine ausreichende Evidenz zu weiteren harten Endpunkten und zum langfristigen Wirkungsgrad.

## Supplementary Information

Tabelle e1 SCALE-Programm (Studien und Ergebnisse für Liraglutid), Tabelle e2 STEP-Programm (Studien und Ergebnisse für Semaglutid), Tabelle e3 Details der unerwünschten Ereignisse unter Liraglutid‑, Semaglutid- und Tirzepatid-Behandlung
