# Supplementary material for: Peptide hormone analogue-based pharmacotherapy for obesity is effective: What is the evidence on hard end points and the long-term course?
Source: Chirurgie (Heidelb). 2025 Nov 5;97(1):11–20. [Article in German] doi: 10.1007/s00104-025-02402-z (PMC12804224; doi:10.1007/s00104-025-02402-z)
Supplement: Supplementary file 1 — Tabelle e1 SCALE-Programm (Studien und Ergebnisse für Liraglutid), Tabelle e2 STEP-Programm (Studien und Ergebnisse für Semaglutid), Tabelle e3 Details der unerwünschten Ereignisse unter Liraglutid‑, Semaglutid- und Tirzepatid-Behandlung [file 104_2025_2402_MOESM1_ESM.pdf]

Online-Supplement

Tabelle e1: SCALE Programm (Studien und Ergebnisse für Liraglutid)

Studie	Ergebnisse nach 56 Wochen (MW ±SD)	Liraglutid 3.0mg N=2487	Placebo N=1244	Behandlungseffekt Liraglutid vs Placebo (95% CI)	p-Wert
**Scale 1 Prä-Diabetes** [6]	Ausgangsgewicht in kg	106.2±21.2	106.2±21.7	-	-
	Gewichtsabnahme %	−8.0±6.7	−2.6±5.7	−5.4 (−5.8 to −5.0)	<0.001
	Gewichtsabnahme in kg	−8.4±7.3	−2.8±6.5	−5.6 (−6.0 to −5.1)	<0.001
	Gewichtsverlust von ≥5% (%)	63.2	27.1	4.8 (4.1 to 5.6)	<0.001
	Gewichtsverlust von ≥10% (%)	33.1	10.6	4.3 (3.5 to 5.3)	<0.001
	Veränderung des HbA1c %	−0.30±0.28	−0.06±0.30	−0.23 (−0.25 to −0.21)	<0.001

Studie	Ergebnisse nach 56 Wochen	Liraglutid 3.0mg n=412	Liraglutid 1.8mg n=201	Placebo n=211	Behandlungseffekt für Liraglutid (95%CI) 3.0mg vs Placebo	p-Wert	1.8mg vs Placebo	p-Wert
**Scale 2 Diabetes NIDDM** [7]	Ausgangsgewicht in kg (SD)	105.7 (21.9)	105.8 (21.0)	106.5 (21.3)	-			-
	Ausgangs HbA1c % MW (SD)	7.9 (0.8)	8.0 (0.8)	7.9 (0.8)	-			-
	Gewichtsabnahme %	–6.0	–4.7	–2.0	4.00 (-5.10-2.90)	<0.001	2.71 (–4.00 – 1.42)	<0.001
	Gewichtsabnahme ≥5%, %	54.3	40.4	21.4	32.9 (24.641.2)	<0.001	19.0 (9.1- 28.8)	<0.001
	Gewichtsabnahme >10%, %	25.2	15.9	6.7	18.5 (12.7 to 24.4)	<.001	9.3 (2.7 to 15.8)	0.006
	Veränderung des HbA1c % MW(SD)	–1.3 (0.9)	–1.1 (1.0)	–0.3 (0.9)	–0.93 (–1.08 – 0.78)	<.001	–0.74 (–0.91 – 0.57)	<.001

Studie	Ergebnisse nach 56 Wochen (MW ±SD)	Liraglutid 3.0mg N=198	Placebo N=198	Behandlungseffekt (95% CI)	p-Wert
**SCALE 3 Diabetes IDDM** [8]	Ausgangsgewicht in kg (SD)	100.6 (20.8)	98.9 (19.9)	N/A	
	Gewichtsabnahme %	-5.8	-1.5	-4.3(-5.5; -3.2)	<0.0001
	Gewichtsabnahme ≥5% (%)	51.8	24.0	3.4 (2.2; 5.3)	<0.0001
	Gewichtsabnahme ≥10% (%)	22.8	6.6	4.2 (2.2; 8.2)	<0.0001
	Veränderung des HbA1c in %	-1.1	-0.6	-0.5 (-0.8; -0.3)	<0.0001
	Veränderung des FPG, mmol/L	-0.1	-0.6	-0.4 (-0.9; 0.1)	0.1502
	Veränderung der täglichen Insulindosis (units)	2.8	17.8	-15.0 (-22.0; -8.0)	<0.0001

	Ergebnisse	Liraglutid 3.0 mg n=212	Placebo n=210	Behandlungseffekt (ETD) oder Odds ratio (OD) Liraglutid v. Placebo (95% CI)	p-Wert
**SCALE 5 Erhaltung des Gewichts-verlusts** [10]	Ausgangsgewicht in kg (SD)	100.4 (20.8)	98.7 (21.2)	-	-
	Gewichtsverlust (kg) nach Diät	-6.3 (1.5)	-6.3 (1.6)	-	-
	**Veränderungen (Randomisierung bis Woche 56)**	**n=207**	**n=206**	-	-
	Gewichtsverlust (%)	-6.2 (7.3)	-0.2 (7.0)	ETD; -6.1 (-7.5 to -4.6)	<0.0001
	Gewichtsverlust >5%	81.4%	48.9%	OR: -4.8 (3.0 to 7.7)	<0.0001
	Gewichtsverlust >10%	50.5%	21.8%	OR: -3.9 (2.4 to 6.1),	<0.0001
	**Veränderungen (Randomisierung bis Woche 68)**	**n=159**	**n=144**	-	-
	Gewichtsverlust (%)	-4.1 (8.2)	0.3 (7.7)	ETD: -4.2 (-6.0 to -2.4)	<0.0001

Tabelle e2: STEP Programm (Studien und Ergebnisse für Semaglutid)

Studie	Ergebnisse nach 68 Wochen	Semaglutid 2.4mg, n=1306	Placebo n=655	Behandlungseffekt oder Odds ratio (95% CI) p-Wert
**STEP 1 Überge-wicht und Adipositas** [11]	Ausgangsgewicht in kg (SD)	105.4 ± 22.1	105.2±21.5	-
	Gewichtsverlust (%)	-14.85	-2.41	ETD –12.44 (-13.37 to -11.51) p<0.001
	Gewichtsverlust (kg)	-15.3	-2.6	ETD -12.7 (-13.7 to -11.7)
	Gewichtsverlust ≥5% (%)	86.4	31.5	OR 11.2 (8.9 to 14.2) p<0.001
	Gewichtsverlust >10% (%)	69.1	12.0	OR 14.7 (11.1 to 19.4) p<0.001
	Gewichtsverlust >15% (%)	50.5	4.9	OR 19.3 (12.9 to 28.8) p<0.001

Studie	Ergebnisse	Baseline		Woche 68		Woche 120	
		Semaglutid	Placebo	Semaglutid	Placebo	Semaglutid	Placebo
**STEP 1 Ver-längerung** [12]	n	228	99	228	99	197	93
	Ausgangs-gewicht in kg (SD)	105.6 ± 21.8	105.4 ± 25.6	87.5 ± 21.4	103.2 ± 25.6	99.0 ± 22.5	105.5 ± 26.2
	Body Mass Index in kg/m2 (SD)	37.6 ± 7.0	37.7 ± 8.0	31.2 ± 7.2	36.9 ± 8.0	35.0 ± 7.1	37.6 ± 8.2
	HbA1c in % (SD)	5.7 ± 0.3	5.7 ± 0.3	5.2 ± 0.3	5.5 ± 0.4	5.6 ± 0.3	5.7 ± 0.5

Studie	Ergebnisse	Semaglutid (2.4mg= fortgesetzt n=535	Wechsel zu Placebo n=268	Behandlungseffekt (95% CI) p-Wert
**STEP 4 Therapie-abbruch**	Ausgangsgewicht in kg (SD), **Woche 0**	107.2 (22.7)		-
	Körpergewicht in kg (SD), **Woche 20**	96.5 (22.5)	95.4 (22.7)	-
	**Ergebnisse von Woche 20 bis Woche 68**			

[13]	Körpergewicht in % (95% CI)	−7.9 (−8.6 to −7.2)	6.9 (5.8 to 7.9)	−14.8 (−16.0 to −13.5) p<0.001
	Körpergewicht in kg (95% CI)	−7.1 (−7.8 to −6.5)	6.1 (5.1 to 7.0)	−13.2 (−14.3 to −12.0) p<0.001
	Body Mass Index in kg/m2 (95% CI)	−2.6 (−2.8 to −2.4)	2.2 (1.8 to 2.5)	−4.7 (−5.2 to −4.3) p<0.001

**Studie**	**Ergebnisse**<br>nach 68 Wochen	**Semaglutid**		**Placebo**	**Behandlungseffekt**<br>**EDT oder OR (95%CI) p-Wert**		
		2.4mg<br>**N=404**	1.0mg<br>**N=403**	**N=403**	Semaglutid 2.4 mg vs Placebo	Semaglutid 2.4 vs 1.0 mg Semaglutid	Semaglutid 1.0 mg vs Placebo
**STEP 2 NIDDM**<br>[14]	Ausgangsgewicht in kg (SD)	99.9 (22.5)	99.0 (21.1)	100.5 (20.9)	-	-	-
	Gewichtsver-änderung in % (SE)	−9.64% (0.4)	−6.99% (0·4)	−3.42% (0·4)	ETD −6.21 (−7·28 to −5·15) p<0·0001	ETD −2.65 (−3·66 to −1·64) p<0·0001	N/A
	Gewichtsver-änderung in kg (SE)	−9·7 (0·4)	−6·9 (0·4)	−3·5 (0·4)	ETD −6·1 (−7·2 to −5·0)	ETD −2·7 (−3·8 to −1·7)	N/A
	Gewichtsverlust ≥5% (%)	68·8	57·1	28·5	OR 4·88 (3·58 to 6·64) p<0·0001	OR 1·62 (1·21 to 2·18)	N/A
	Veränderung des HbA1c % (SE)	−1·6 (0·1)	−1·5 (0·1)	−0·4 (0·1)	ETD −1·2 (−1·4 to −1·0) p<0·0001	ETD −0·2 (−0·3 to 0·0)	ETD −1·1 (−1·3 to −0·9)

**Studie**	**Ergebnisse**<br>nach 68 Wochen	**Semaglutid 2.4mg**<br>**n=199**	**Semaglutid 1.7mg**<br>**n=101**	**Placebo**<br>**n=101**
**STEP 6 NIDDM**<br>[15]	Ausgangsgewicht in kg (SD)	86.9 (16.5)	86.1 (11.9)	90.2 (15.1)
	Type 2 Diabetiker n (%)	49/199 (25%)	25/101 (25%)	25/101 (25%)
	Ausgangs HbA1c % (SD)	8.4 (0.8)	8.2 (0.7)	8.1 (0.8)
	Gewichtsverlust (SEM) %<br><br>Behandlungseffekt Semaglutid vs. Placebo (95%CI) p-Wert	–13.2% (0.5)<br><br>–11.06 (–12.88 –9.24) p<0.0001	–9.6% (0.8)<br><br>–7.52 (–9.62 –5.43) p<0.0001	–2.1% (0.8)<br><br>-
	Gewichtsverlust ≥5% (%)<br><br>Odd ratio Semaglutid vs. Placebo (95%CI) P value	83<br><br>21.72 (11.27- 41.86) p<0.0001	72<br><br>11.08 (5.53- 22.22) p<0.0001	21<br><br>-
	Veränderung des HbA1c %	–2·2%	–2·1%	0.3

**Studie**	**Ergebnisse nach 44 Wochen**	**Semaglutid 2.4mg**<br>**n=249**	**Placebo**<br>**n=126**	**ETD/OR (95%CI)**<br>**p-Wert**
**STEP 7 IDDM**<br>[16]	Ausgangsgewicht in kg (SD)	96.4 (17.9)	96.2 (17.3)	-
	Type 2 Diabetiker, n (%)	64 (26%)	32 (25%)	-
	Ausgangs HbA1c % (SD)	8.0 (0.7)	8.2 (0.9)	
	Gewichtsverlust (SEM) %	–12.1% (0.5)	–3.6% (0.7)	ETD –8.5 (–10.2 to –6.8) P<0.0001
	Gewichtsverlust ≥5% (%)	85	31	OR 13.1 (7.4 to 23.1) P<0.0001
	Veränderung des HbA1c %	–0.8 (0.1)	–0.1 (0.1)	ETD –0.7 (–0.8 to –0.5) P<0.0001

**Studie**	**Ergebnisse nach 68 Wochen**	**Semaglutid 2.4mg**<br>**n=407**	**Placebo**<br>**n=204**	**ETD/Odds ratio (95%CI) p-Wert**
**STEP 3**	Ausgangsgewicht in kg (SD)	106.9 (22.8)	103.7 (22.9)	-
	Gewichtsverlust (SEM) in %	–16.0	–5.7	ETD –10.3 (–12.0 to –8.6) p<0.001
	Gewichtsverlust ≥5% in %	86.6	47.6	OR 6.1 (4.0 to 9.3) p<0.001

**Intensive Diät** [17]	Gewichtsverlust ≥10% in %	75.3	27.0	OR 7.4 (4.9 to 11.0) p<0.001
	Gewichtsverlust ≥15% in %	55.8	33.2	OR 7.9 (4.9 to 12.6) p<0.001
	Gewichtsverlust in kg	–16.8	–6.2	ETD –10.6 (–12.5 to –8.8) p<0.001
**Studie**	**Ergebnisse nach 104 Wochen**	**Semaglutid 2.4mg n=152**	**Placebo, n=152**	**ETD / OR (95% CI) p-Wert**
**STEP 5 2 Jahre** [18]	Ausgangsgewicht in kg (SE)	105.6 (20.8)	106.5 (23.1)	-
	Gewichtsverlust nach 104 Wochen (SEM) in %	–15.2 (0.9)	–2.6 (1.1)	ETD –12.6 (–15.3 to –9.8) *p*<0.0001
	Gewichtsverlust nach 52 Wochen (SEM) in %	–15.6 (0.7)	–3.0 (0.7)	ETD –12.6 (–14.5 to –10.7) *p*<0.0001
	Gewichtsverlust nach 104 Wochen (SEM) in kg	–16.1 (1.0)	–3.2 (1.2)	ETD –12.9 (–16.1 to –9.8) *p*<0.0001
	Gewichtsverlust ≥5% nach 104 Wochen in %	77.1	34.4	OR 5.0 (3.0 to 8.4) *P* < 0.0001
	Gewichtsverlust ≥10% nach 104 Wochen in %	61.8	13.3	OR 7.2 (4.0 to 13.2) *P* < 0.0001
**Studie**	**Ergebnisse nach 68 Wochen**	**Semaglutid 2.4ng n=126**	**Liraglutid 3.0mg n=127**	**ETD / Odds Ratio (95%CI) p-Wert**
**STEP 8 Sema- vs. Liraglutid** [19]	Ausgangsgewicht in kg (SD)	102.5 (25.3)	103.7 (22.5)	-
	Gewichtsverlust in % (95% CI)	–15.8 (–17.6 to –13.9)	–6.4 (–8.2 to –4.6)	ETD–9.4 (–12.0 to –6.8) p<0.001
	Gewichtsverlust von ≥10% in %	70.9	25.6	6.3 (3.5 to 11.2) p<0.001
	Gewichtsverlust von ≥15% in %	55.6	12.0	7.9 (4.1 to 15.4) p<0.001
	Gewichtsverlust von ≥20% in %	38.5	6.0	8.2 (3.5 to 19.1) p<0.001
	Gewichtsverlust in kg, (95%CI)	–15.3 (–17.3 to –13.4)	–6.8 (–8.8 to –4.9)	–8.5 (–11.2 to –5.7) p<0.001
**Studie**	**Ergebnisse**	**Semaglutid 2.4mg n=138**	**Placebo n=69**	**ETD/OR (95%CI) p-Wert**
**STEP 10 Prädiabetes** [21]	Ausgangsgewicht in kg (SD)	111.9 (21.5)	111.0 (23.5)	-
	Ausgangs-HbA1c % (SD)	5.9% (0.3)	5.9% (0.3)	-
	Ausgangs FPG, mmol/L (SD)	5.8 (0.5)	6.0 (0.7)	
	Gewichtsverlust in % (SD)	–13.9% (0.7)	–2.7% (0.6)	ETD –11.2 (–13.0 –9.4) p<0.0001
	Anteil derer, die eine Normoglykämie erreichten (HbA1c <6.0%, FPG <5.5mmol/L) in %	81	14	OR 19.8 (8.7 - 45.2) p<0.0001
	Gewichtsverlust in kg (SD)	–15.2 (0.8)	–2.8 (0.6)	ETD –12.4 (–14.4 –10.3) p<0.0001
	Änderung des HbA1c in % (SD)	–0.4% (0.3)	0.1% (0.3)	STD –0.5% (–0.5 – 0.4) p<0.0001
	Änderung der FPG in mmol/L (SD)	–0·8 (0·1)	–0·2 (0·1)	–0.6 (–0.8 – 0.4) p<0.0001
	Anteil derer mit Progression zu einem Typ 2 Diabetes nach 52 Wochen in %	1	3	-

Tabelle e3: Details der unerwünschten Ereignisse unter Liraglutid, Semaglutid und Tirzepatid Behandlung

Programm	Studie	Behandlung	Erkrankungen der Gallenblase		Pankreatitis		Medulläres Schilddrüsen-karzinom		Suizidalität		Gastrointestinale Ereignisse	
			%	Ereignisrate pro 100 Behandlungs-jahren	%	Ereignisrate pro 100 Behandlungs-jahren	%	Ereignisrate pro 100 Behandlungs-jahren	%	Ereignisrate pro 100 Behandlungs-jahren	%	Ereignisrate pro 100 Behandlungs-jahren
**SCALE Programm**	**SCALE 1** [6]	Liraglutid 3.0mg n=2487	2.5	3.1	0.2	0.2	0	-	0	-	-	
		Placebo n=1224	1.0	1.4	0	0	0	-	0	-	-	
	**SCALE 2** [7]	Liraglutid 3.0mg n=422	1.2	-	0	0	0	-	0	-	65.2	224
		Liraglutid 1.8mg n=210	1.9	-	0	0	0	-	0	-	56.2	148
		Placebo n=212	0.5	-	0	0	0	-	0	-	39.2	83
	**SCALE 3** [8]	Liraglutid 3.0mg n=195	1.0	-	0	-	0	-	3.5	-	62.1	207.1
		Placebo n=197	0.5	-	0.5	-	0	-	4.0	-	46.7	101.9
	**SCALE 4** [9]	Liraglutid 3.0mg n=176	0.6	1.1	0	-	0	-	0.6	1.1	-	-
		Placebo, n=179	0.6	1.0	0	-	0	-	0	-	-	-
	**SCALE 5** [10]	Liraglutid 3.0mg n=212	-	-	0	-	0	-	2.3	-	73.6	254.5
		Placebo n=210	-	-	0	-	0	-	1.9	-	45.2	98.6
	**STEP 1** [11]	Semaglutid 2.4mg n=1306	2.6	2.5	0.2	0.2	-	-	-	-	74.2	252.6
		Placebo n=655	1.2	1.0	0	-	-	-	-	-	47.9	89.1
	**STEP 2** [14]	Semaglutid 2.4mg n=403	0.2	0.4	0.2	0.3	0	-	-	-	63.5	173.3
		Semaglutid 1.0mg n=402	1.0	0.8	0	-	0	-	-	-	57.5	136.7
		Placebo n=402	0.7	0.8	0.2	0.2	0	-	-	-	34.3	49.6
	**STEP 3** [17]	Semaglutid 2.4mg n=407	4.9	4.6	0	-	0	-	-	-	82.8	334.5
		Placebo n=204	1.5	1.1	0	-	0	-	-	-	63.2	127.4
	**STEP 4**	Semaglutid 2.4mg n=535	2.8	3.1	0	-	-	-	-	-	41.5	111.9

	[13]	Placebo n=268	3.7	4.1	0	-	-	-	-	-	26.1	46.6
**STEP Programm**	**STEP 5** [18]	Semaglutid 2.4mg n=152	2.6	2.0	0	-	-	-	-	-	82.2	230.7
		Placebo n=152	1.3	0.7	0	-	-	-	-	-	53.9	94.1
	**STEP 6** [15]	Semaglutid 2.4mg n=199	1.0	0.7	0	-	0	-	-	-	59.0	124.7
		Semagluitd 1.7mg n=100	1.0	0.7	0	-	0	-	-	-	64.0	187.9
		Placebo n=101	1.0	0.7	0	-	0	-	-	-	30	30.3
	**STEP 7** [16]	Semaglutid 2.4mg n=249	2	2.2	0	-	-	-	-	-	67	203
		Placebo n=126	2	2.6	0	-	-	-	-	-	36	75.3
	**STEP 8** [19]	Semaglutid 2.4mg n=126	0.8	-	0	-	0	-	-	-	84.1	-
		Liraglutid 3.0mg n=127	3.1	-	0.8	-	0	-	-	-	82.7	-
		Placebo n=85	1.2	-	0	-	0	-	-	-	55.3	
	**STEP 9** (nur SAEs) [20]	Semaglutid 2.4mg n=269	1.1	-	0	-	0	-	-	-	1.5	-
		Placebo n=135	0.7	-	0	-	0	-	-	-	0.7	-
	**STEP 10** (nur SAEs) [21]	Semaglutid 2.4mg n=138	1	0.7	1	2.9	0.7	-	-	-	2	5.7
		Placebo n=69	0	0	0	0	0	-	-	-	0	0
**SELECT** (Nur SAEs) [29]		Semagluitd 2.4mg n=8803	2.8	-	0.2	-	-	-	-	-	3.9	-
		Placebo n=8801	2.3	-	0.3	-	-	-	-	-	3.7	-
	**SURMOUNT 1** [22]	Tirzepatid 15mg n=630	1.0	-	0.2	-	-	-	-	-	-	-
		Tirzepatid 10mg n=636	1.7	-	0.2	-	-	-	-	-	-	-
		Tirzepatid 5mg, n=630	0.8	-	0.2	-	-	-	-	-	-	-
		Placebo n=643	0.8	-	0.2	-	-	-	-	-	-	-
	**SURMOUNT 1 Ext** [23]	Tirzepatid 15mg,n=253	3.6	-	0	-	0	-	0	-	2.4	-
		Tirzepatid 10mg,n=262	3.8	-	0.4	-	0	-	0.4	-	5.7	-
		Tirzepatid 5mg,n=247	1.2	-	0.8	-	0	-	0	-	2.4	-
		Placebo n=270	0.7	-	0.4	-	0	-	0	-	1.9	-
	**SURMOUNT 2** (nur SAEs) [25]	Tirzepatid 10mg,n=312	1.0	-	0	-	0	-	-	-	2	-
		Tirzepatid 15mg,n=311	1.0	-	1	-	0	-	-	-	3	-

**SURMOUNT program**		Placebo n=315	1.0	-	<1	-	0	-	-	-	1	-
	**SURMOUNT 3** (nur SAEs)[26]	Tirzepatid MTD, n=287	0.7	-	0.3	-	0	-	0.3	-	5.6	-
		Placebo n=292	0	-	0.3	-	0	-	0	-	1.7	-
	**SURMOUNT 4** (serious events) [27]	Tirzepatid MTD, n=335	0	-	0	-	0	-	0	-	1.8	-
		Placebo n=335	0.9	-	0	-	0	-	0	-	0.3	-
	**SURMOUNT 5** nur SAEs) [28]	Tirzepatid MTD, n=374	1.1	-	0	-	0	-	0	-	4.5	-
		Semaglutid MTD, n=376	1.3	-	0.3	-	0	-	0	-	3.7	-
	**SURMOUNT J** [24]	Tirzepatid 10mg, n=73	0	-	0	-	0	-	0	-	-	-
		Tirzepatid 15mg, n=77	1.0	-	0	-	0	-	0	-	-	-
		Placebo n=75	0	-	0	-	0	-	0	-	-	-
